# Specific metallo-protein interactions and antimicrobial activity in Histatin-5, an intrinsically disordered salivary peptide

**DOI:** 10.1038/s41598-019-52676-7

**Published:** 2019-11-21

**Authors:** Tyler G. McCaslin, Cynthia V. Pagba, Jiby Yohannan, Bridgette A. Barry

**Affiliations:** 1 School of Chemistry and Biochemistry, Georgia Institute of Technology, Atlanta, GA 30332 USA; 2 The Parker H. Petit Institute of Bioengineering and Bioscience, Georgia Institute of Technology, Atlanta, GA 30332 USA

**Keywords:** Biophysical chemistry, Biochemistry

## Abstract

Histatin-5 (Hst-5) is an antimicrobial, salivary protein that is involved in the host defense system. Hst-5 has been proposed to bind functionally relevant zinc and copper but presents challenges in structural studies due to its disordered conformation in aqueous solution. Here, we used circular dichroism (CD) and UV resonance Raman (UVRR) spectroscopy to define metallo-Hst-5 interactions in aqueous solution. A zinc-containing Hst-5 sample exhibits shifted Raman bands, relative to bands observed in the absence of zinc. Based on comparison to model compounds and to a family of designed, zinc-binding beta hairpins, the alterations in the Hst-5 UVRR spectrum are attributed to zinc coordination by imidazole side chains. Zinc addition also shifted a tyrosine aromatic ring UVRR band through an electrostatic interaction. Copper addition did not have these effects. A sequence variant, H18A/H19A, was employed; this mutant has less potent antifungal activity, when compared to Hst-5. Zinc addition had only a small effect on the thermal stability of this mutant. Interestingly, both zinc and copper addition shifted histidine UVRR bands in a manner diagnostic for metal coordination. Results obtained with a K13E/R22G mutant were similar to those obtained with wildtype. These experiments show that H18 and H19 contribute to a zinc binding site. In the H18A/H19A mutant the specificity of the copper/zinc binding sites is lost. The experiments implicate specific zinc binding to be important in the antimicrobial activity of Hst-5.

## Introduction

Intrinsically-disordered proteins (IDPs) play key roles in human health (reviewed in refs.^1–3^). IDPs are inherently difficult to study, because their dynamic conformational landscapes interconvert between different structures on a variety of timescales^4–6^. Histatins are a class of histidine-rich salivary peptides, which are important in the innate immune system and play an antimicrobial role (reviewed in^7^). These peptides are antimicrobial against various bacteria and fungi such as multi-drug-resistant *Staphylococcus aureus *(MRSA)^8^, *Candida albicans*^9,10^, *Cryptococcus neoformans*^11^, and can regulate *Porphyromonas gingivalis*, which is associated with periodontal disease^12^. Amongst the histatin family, the two primary sequences are Hst-1 and Hst-3, which are genetically encoded by the *his1* and *his2* genes, respectively^13^. Hst-5 is proposed to be a 24-amino acid proteolytic product of Hst-3^13^. Hst-2 is likely derived from Hst-1, while Hst-4 through Hst-12 are likely fragments of Hst-3^14^. Hst-1 has been shown to undergo post-translational modifications. Ser2 phosphorylation has been identified in Hst-1^15^, and polysulfation of the terminal four tyrosines (Tyr27, 30, 34, and 36) of Hst-1 has been detected^16^.

**Figure 1:**
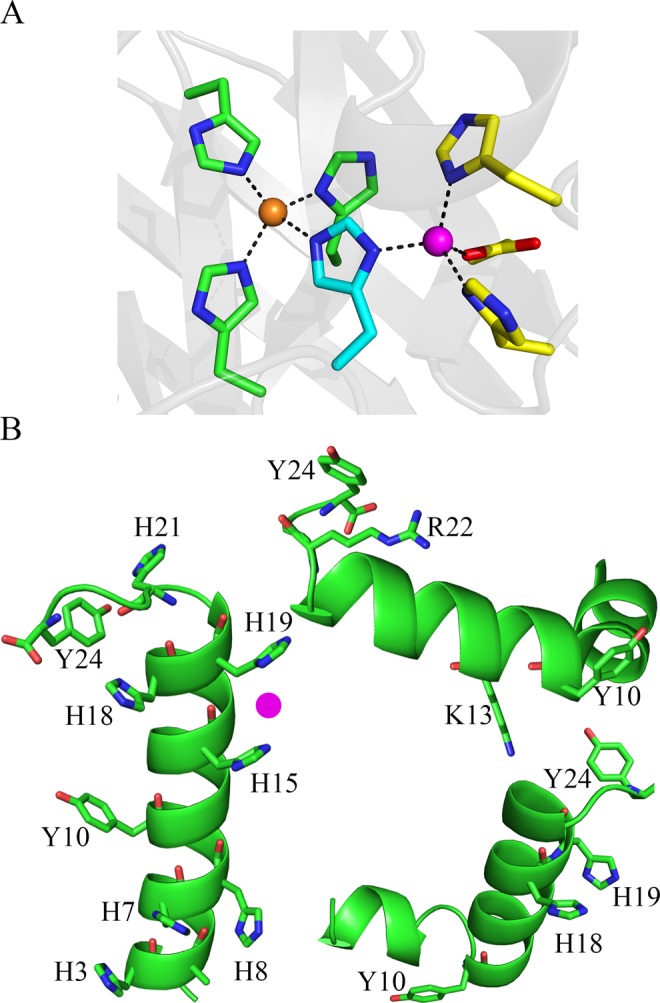
In (**A**), copper/zinc coordination site in superoxide dismutase (SOD) (PDB, 2SOD)^57^. Cu^2+^ (light brown sphere) is ligated by His44, His46, His61 and His118, while Zn^2+^ (magenta sphere) is ligated by His61, His69, His78 and Asp81. His61 (blue residue) bridges Cu^2+^ and Zn^2+^ ions. In (**B**), speculative PEP-FOLD models of Hst-5. Side chain labeling: left, all seven histidine and both tyrosine side chains are labeled; top, K13, R22, and both tyrosine residues; bottom right, H18, H19, and both tyrosines are labeled. The mutants, Hst-5 K13E/R22G (top) and H18A/H19A (bottom) are investigated here. A speculative binding site for the Zn^2+^ ion is shown in pink.

**Figure 2:**
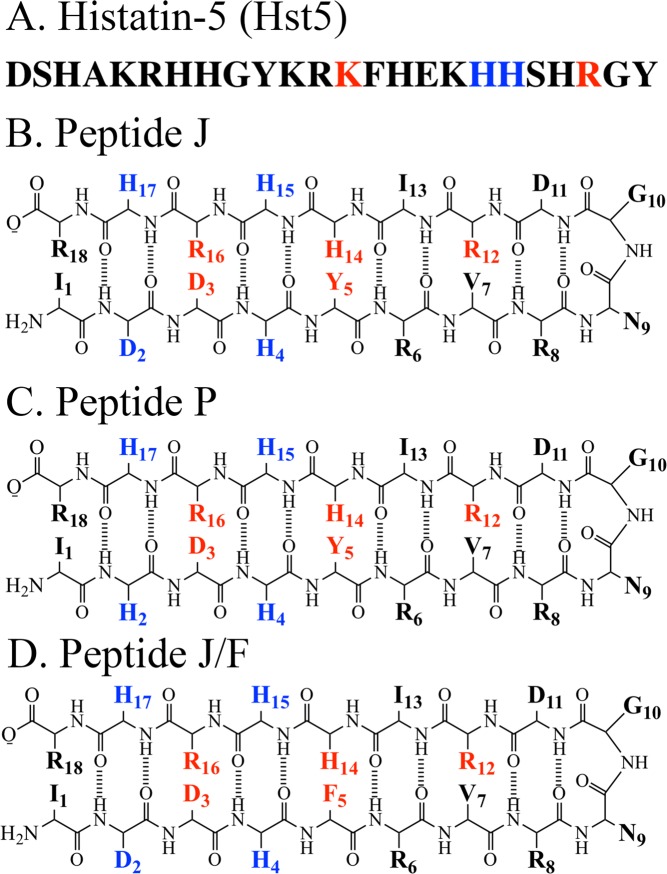
Sequences of Hst-5 (**A**), Peptide J (**B**), Peptide P (**C**), and Peptide J/F (**D**). In (**A**), red denotes K13E/R22G mutation sites and blue denotes H18A/H19A mutation sites. In (B-D), blue denotes metal-binding residues and red denotes possible non-covalent interactions with Tyr5.

The focus of this study is Hst-5 (Figs. 1 and 2A). In aqueous solution, the conformation of Hst-5 is disordered^17^. However, in trifluoroethanol^18^ or d_6_-DMSO^19^, Hst-5 adopts a helical conformation. In particular, studies of Hst-5 and synthetic analogs have shown that the CD and NMR signals are characteristic of a disordered protein. For example, there is no defined near-UV CD signal in synthetic analogs of Hst-5^20^. Small angle X-ray scattering and Monte-Carlo simulations have been used to explain the effects of pH and ionic strength on Hst-5^21–24^.

Within this family of peptides, Hst-5 has been proposed to bind functionally relevant Cu^2+^ and Zn^2+^ ^7,18,20^. Copper-zinc binding sites are found in enzymes, such as superoxide dismutase (Fig. 1A) and can consist of histidine side chains (reviewed in ref.^25^). Cu^2+^ binding by Hst-5 has been proposed to occur and to involve the amino terminus^26^. EPR spectroscopy has been used to investigate the copper binding site^27^. Copper has been proposed to provide oxidative activity and may be responsible for the antimicrobial properties of Hst-5^20,27,28^. Nickel has been shown to bind to Hst-5, and may implicate Hst-5 in nickel allergies^29^. Zinc interactions have also been proposed to enhance antimicrobial activity^30^ and promote the formation of highly dynamic oligomers^24^.

To summarize, the functional and structural role of metal binding is important in clinical applications of salivary peptides and requires additional clarification. In this work, we use circular dichroism (CD) and UV resonance Raman (UVRR) spectroscopy to obtain new information concerning the structure and function of Hst-5 and its interactions with zinc and copper. In addition to wildtype, two mutants of Hst-5 were selected for study, K13E/R22G and H18A/H19A (Figs. 1 and 2A). Compared to the wild type sequence (ED_50_ = 6–8 μM), K13E/R22G has approximately 7-fold less potency (ED_50_ = 50 μM) as a candidacidal agent^10^. In the second mutant, HstH18A/H19A, this sequence has approximately 20-fold less potency (ED_50_ = 150 μM) against *Candida*^9^. It has been shown previously that synthetic Hst-5 exhibits similar candidacidal activity to the native peptide^31^.

Because Hst-5 is intrinsically disordered, spectroscopic results represent the properties of a dynamic ensemble. To interpret results, comparison to model amino acids in is an important first step. In this paper, we also compare Hst-5 results to data acquired from a novel family of beta hairpin peptides. The beta hairpins contain two antiparallel beta strands connected by a turn motif^32,33^. The peptides are derivatives of a parent sequence, Peptide A, which does adopt a defined structure^32–35^. The structures of Peptide A and some of its sequence variants have been solved by NMR spectroscopy^32^. However, the peptides employed here, called Peptide P, Peptide J, and Peptide J/F (Fig. 2B–D), are Peptide A modifications that have no defined structure in solution. The peptides contain either a four histidine (Peptide P) or a three histidine-one aspartate (Peptide J and Peptide J/F) metal coordination site. The Peptide J/F sequence has been altered to contain a phenylalanine instead of the tyrosine found in Peptide J. The coordination sites engineered into Peptides P, J, and J/F were designed to bind zinc. Zinc interactions in proteins and peptides are particularly challenging to study because zinc is non-chromogenic and non-paramagnetic. Our experiments provide new information concerning metal interactions and structure and function in conformationally flexible proteins such as Hst-5.

## Results

### UV Vis and CD spectroscopy

Hst-5 was synthesized by solid state synthesis (Fig. 2A). Though Hst-5 is intrinsically disordered, PEP-FOLD^36^ models of the wild type sequence were generated and are shown as speculative examples of possible structures in Fig. 1B. Three different models are shown and display amino acid side chains of relevance to this study, namely Tyr10, Tyr24, His3, His7, His8, His15, His18, His19, His21, Lys13, and Arg22.

**Figure 3:**
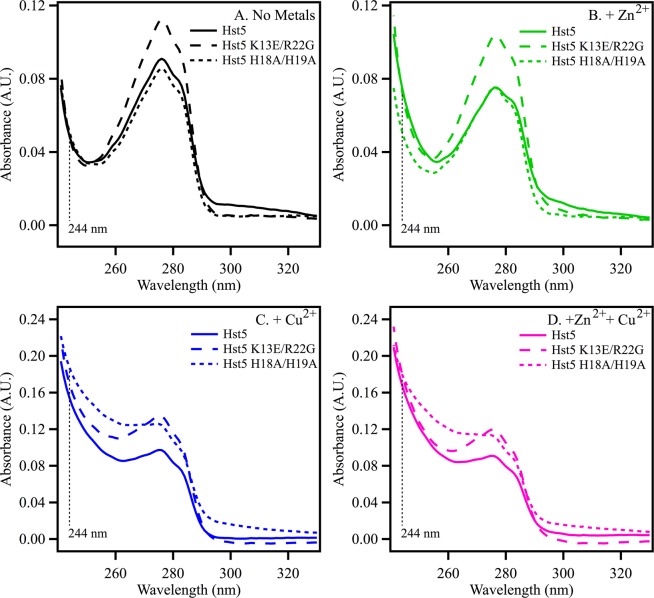
UV absorption spectra of Hst-5 and mutants. In (A**)**, Hst-5 (solid line), K13E/R22G (dashed line), and H18A/H19A (dotted line) in the absence of added metals (black). In (**B**), Hst-5 (solid line), K13E/R22G (dashed line), and H18A/H19A (dotted line) in the presence of equimolar Zn^2+^ (green). In (**C**), Hst-5 (solid line), K13E/R22G (dashed line), and H18A/H19A (dotted line) in the presence of equimolar Cu^2+^(blue). In (**D**), Hst-5 (solid line), K13E/R22G (dashed line), and H18A/H19A (dotted line) in the presence of equimolar Zn^2+^ and Cu^2+^. The peptide concentration was 50 μM, and the buffer contained 5 mM HEPES, pH 7.5 and 50 μM ZnCl_2_ and/or CuCl_2_ where noted and the metal- or non-metal-containing buffers were used in a reference cell during spectrum acquisition. Spectra are an average of three measurements; resolution, 1 nm; tick marks denote 0.04 absorbance units.

Hst-5 contains an amino terminal copper and nickel binding motif as well as a proposed binding site for zinc (HEXXH)^7^ corresponding to H_15_EKH_18_ in Hst-5. The peptide contains two tyrosines and seven histidines, and shows absorbance in the UV region of the spectrum (Fig. 3A, solid line). The addition of Zn^2+^ (Fig. 3B), Cu^2+^ (Fig. 3C) or a mixture of Zn^2+^ and Cu^2+^ (Fig. 3D) alters the spectrum of Hst-5.

**Figure 4:**
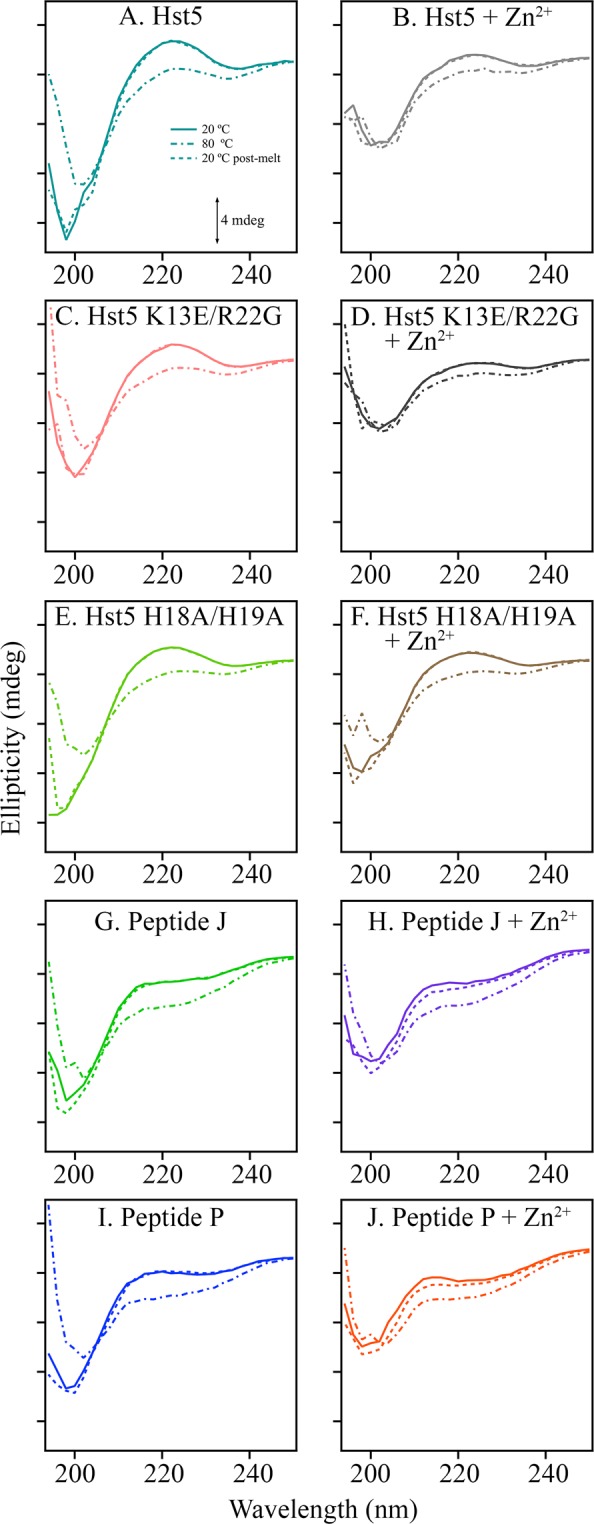
CD spectra of peptides in the absence (left) or presence of (right) Zn^2+^. Samples are Hst-5 (**A**, teal), Hst-5 + Zn^2+^ (**B**, grey), K13E/R22G (**C**, peach), K13ER22G + Zn^2+^ (**D**, black), H18A/H19A (**E**, light green), H18A/H19A + Zn^2+^ (**F**, brown), Peptide J (**G**, green), Peptide J + Zn^2+^ (**H**, purple), Peptide P (**I**, blue), and Peptide P + Zn^2+^ (**J**, orange). Spectra were acquired at 20 °C (solid trace), then the samples were heated in a Peltier cell at 80 °C (dot-dashed trace), and subsequently cooled back to 20 °C (dashed trace). Tick marks denote 4 mdeg. Spectra were averaged from three to nine replicates. The peptide concentration was 100 μM, and equimolar ZnCl_2_ was added where noted. The buffer contained 5 mM HEPES pH 7.5.

The peptide was characterized by CD experiments and thermal melting. In Fig. 4A (solid line), the pre-melt spectrum at 20 °C (solid line) of Hst-5 shows two features: a band of negative ellipticity at 198 nm and a broad positive feature at approximately 220 nm. With heating to 80 °C, these features are lost (Fig. 4A, dot-dashed line), but regained after cooling (Fig. 4A, dashed line). The addition of zinc in a 1:1 ratio is associated with a less significant change with heating and cooling (Fig. 4B). Such an effect can be associated with zinc-driven stabilization of peptide conformation. Zinc-induced changes to the CD spectrum have been reported previously, although those previously reported spectra are distinguishable from the data derived here^37^. The CD spectrum of Hst-5 in various solvents has been reported ^10,19,37^. Spectra reported previously in aqueous solution are distinguishable from the data derived here^37^. While the CD spectrum is responsive to alterations in secondary structure, the overlap of spectral components can make interpretation challenging in the far-UV^38^. The CD spectrum of Hst-5 shown here has some similarity to reported CD spectra of polyproline helices^38^. Small differences between premelt and postmelt data are not considered to be significant, given the noise in the CD measurements. In the absence of metal ions and defined protein structure, a near-UV CD signal is not expected. See ref.^20^ for an example with a Hst-5 analog^20^.

To interpret these results, a metal binding site was engineered into a family of beta hairpin peptides. The original peptide, Peptide A, is known to be a beta hairpin from NMR spectroscopy^32^. This peptide contains a tyrosine-histidine pair, which conducts a proton coupled electron transfer reaction when tyrosine is oxidized in the mid-pH range^32,33^. As discussed above (Fig. 1A), zinc binding sites in proteins can be composed of histidine and carboxylate groups^39^. Therefore, three types of zinc binding sites were engineered into Peptide A. The first, giving Peptide P, contains a four histidine motif (H2, H4, H15, and H17) (Fig. 2C). The far-UV spectrum of Peptide P is shown in Supporting Fig. S1C (pH 7.5) and S1D (pD 7.5) and represents the contributions of five histidines and one tyrosine, Y5. Difference spectra of Peptide P with varying ratios of peptide to metal were generated (Supporting Fig. S2A,C) and exhibit a red-shifted UV absorption as Zn^2+^ concentration increases (Supporting Fig. S2B,D). The CD spectrum of Peptide P in the absence of zinc exhibits negative ellipticity at 198 nm (Fig. 4I, solid). This band is lost with heating and regained by cooling (Fig. 4I, dot-dashed and dashed). In the presence of zinc (1:1 stoichiometry), the CD spectrum of Peptide P is reduced in amplitude and exhibits only a modest change with an increase in temperature (Fig. 4J). This is consistent with stabilization of peptide structure by the divalent metal ion. Overall, the CD results obtained with Peptide P and zinc are similar to the behavior observed in Hst-5.

Two additional metal binding peptides were characterized with UV absorbance and CD spectroscopy. In Peptide J, the putative metal binding site contains three histidines (H4, H15, H17), with the fourth position occupied by an aspartate (D2) (Fig. 2B). In Peptide J/F, a three-histidine-aspartate binding site is retained (Fig. 2D), but the tyrosine at position 5 is replaced with phenylalanine, which cannot coordinate metals. UV absorbance spectra of Peptide J are presented in Supporting Fig. S1A,B and difference spectra with varying ratios of peptide to metal are shown at pH 7.5 (Supporting Fig. S3A) and pD 7.5 (Supporting Fig. S3C). Again, addition of metal causes a red shift of the Peptide J UV spectrum (Supporting Fig. S3B,D), similar to the response observed in Peptide P (Supporting Fig. S2B,D). CD data derived from Peptide J/F (data not shown) and Peptide J (Fig. 4G,H solid line) exhibit similar behavior when compared to Hst-5 and Peptide P. Note that control CD experiments were performed with Peptide A, which does not contain a metal binding site and has a well defined NMR structure^32^ (Supporting Fig. S4). There was no significant effect of zinc on the stability of the Peptide A beta hairpin. In addition, Peptide P and J samples were frozen, thawed, and filtered before some of the measurements (data not shown). No change was observed. The results support the conclusion that a soluble form of each peptide is interacting with zinc, not an aggregated oligomeric form.

Two mutants of Hst-5 were selected for characterization: K13E/R22G and H18A/H19A (Figs. 1B and 2A, red and blue). These mutants have been shown to exhibit less potent antimicrobial activity^9,10^, when compared to wildtype. The UV spectrum of the mutants in shown in Fig. 3. Note the small change in extinction coefficient at 280 nm in the K13E/R22G mutant (Fig. 3, dashed line), which is most likely associated with an electrostatic perturbation to the environment of one or more of the tyrosines. The spectrum of the tyrosine aromatic ring is known to be sensitive to protonation state and exhibits a red shift when the phenol form is converted to phenolate^34,40^. The CD spectra of the K13E/R22G mutant (Fig. 4C, solid line) are similar to wild type Hst-5; the addition of zinc causes a change in the spectrum consistent with an overall increase in stability, as observed in wildtype (Fig. 4D, solid line). However, in the H18A/H19A mutant (Fig. 4E, solid line), zinc addition has a less significant effect on the CD spectrum (Fig. 4F, solid line) and the thermal melt experiment (Fig. 4E,F, dot-dashed line). The overall affinity for Zn^2+^ binding appears to decrease in the H18A/H19A mutant. The change in the CD signal is consistent with an alteration in the average conformation in the histidine mutant.

### UVRR spectroscopy of Hst-5 and variants

**Figure 5:**
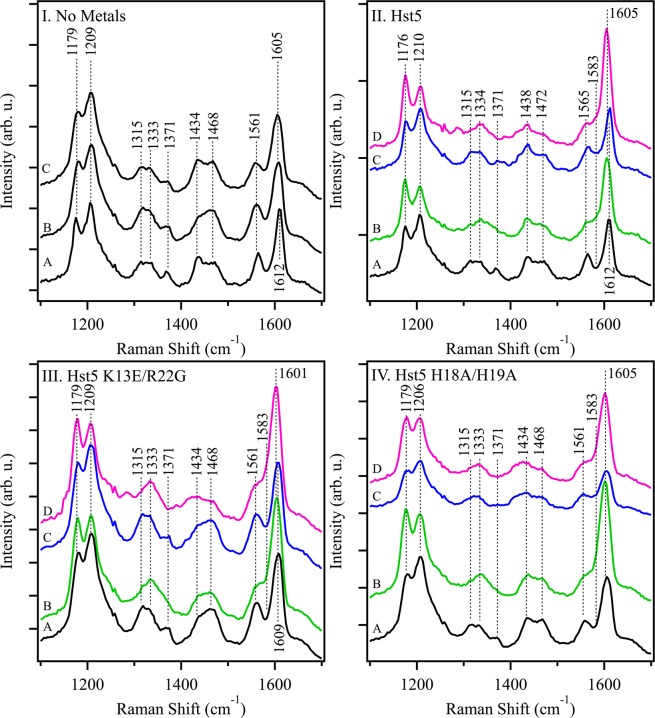
UVRR spectra of Hst-5 and mutants K13E/R22G and H18A/H19A in the presence or absence of Zn^2+^ and Cu^2+^. In (**I**), Hst-5 (A), K13E/R22G (B) and H18A/H19A (C) in the absence of added metals. In (**II**), Hst-5 in the absence of added metals (A, black) and after addition of Zn^2+^ (B, green), Cu^2+^ (C, blue), or a mixture of Zn^2+^ and Cu^2+^ (D, pink). In (**III**), K13E/R22G in the absence of added metals (A, black) and after addition of Zn^2+^ (B, green), Cu^2+^ (C, blue), or a mixture of Zn^2+^ and Cu^2+^ (D, pink). In (**IV**), H18A/H19A in the absence of added metals (A, black) and after addition of Zn^2+^ (B, green), Cu^2+^ (C, blue), or a mixture of Zn^2+^ and Cu^2+^ (D, pink). The peptide concentration was 1 mM, and the samples contained equimolar ZnCl_2_ and/or CuCl_2_ where noted. The buffer contained 5 mM HEPES pD 7.5. The sample was recirculated using a peristaltic pump to prevent UV damage. Laser wavelength, 244 nm; laser power, 3.7 mW; scan time, 120 s; accumulations, 4. Data were averaged from two independent measurements (using two 1 mL samples) and normalized to the intensity of the amide II’ band at ~1434 cm^−1^. Tick marks denote 1000 intensity units.

As a more detailed probe of zinc-peptide interactions, UVRR spectroscopy was employed. In particular, a 244 nm probe and D_2_O buffer allows specific metal interactions with histidine and tyrosine side chains to be observed because the bands are resonantly enhanced at this probe wavelength. The UV spectrum of Hst-5 (Fig. 3, solid line) shows significant absorption at 244 nm. For these UVRR experiments, a microcell recirculating device was used to prevent UV damage to the sample^34,41^. Previous work has shown that the use of D_2_O enhances the vibrational contributions of histidine in the UVRR spectrum^42,43^. Note that the pD reported here is the uncorrected pH meter reading, according to a standard protocol^44^. The UVRR spectrum of Hst-5 in D_2_O solution is presented in Fig. 5IA,IIA and exhibits bands at 1315 cm^−1^, 1334 cm^−1^, 1371 cm^−1^, and 1565 cm^−1^, which are candidates for assignment to the histidine side chain. Bands at 1176 cm^−1^, 1210 cm^−1^, and 1612 cm^−1^ are candidates to be assigned to tyrosine. See Table 1 for a list of observed Raman bands.

**Table 1:** Vibrational bands of aromatic amino acids and comparison to model compounds, peptides and proteins.

Sample	CH bend (Y9a) (cm^−1^)	Ring C-CH_2_ (Y7a) (cm^−1^)	Ring (Y8a) (cm^−1^)			
**Tyrosine**						
Hst-5	1176	1210	1612			
Hst-5 + Zn	1176	1210	1605			
Hst-5 + Cu	1176	1210	1612			
Hst-5 + Zn + Cu	1176	1210	1605			
Hst-5 K13E/R22G	1179	1209	1609			
Hst-5 K13E/R22G + Zn	1179	1209	1601			
Hst5 K13E/R22G + Cu	1179	1209	1609			
Hst-5 K13E/R22G + Zn + Cu	1179	1209	1601			
Hst-5 H18A/H19A	1179	1206	1605			
Hst-5 H18A/H19A + Zn	1179	1206	1605			
Hst-5 H18A/H19A + Cu	1179	1206	1605			
Hst-5 H18A/H19A + Zn + Cu	1179	1206	1605			
Peptide P	1177	1207	1612			
Peptide P + Zn	1177	1207	1604			
Peptide J	1177	1207	1612			
Peptide J + Zn	1177	1207	1604			
Cu/Zn SOD (pD 9, 240 nm ex.)^43^	1177	1206	1616			
**Histidine**						
Sample	~1315 cm^−1^	~1335 cm^−1^	~1370 cm^−1^	~1390 cm^−1^	~1565 cm^−1^	~1580 cm^−1^
Hst-5	1315	1334	1371	N.O.	1565	N.O.
Hst-5 + Zn	N.O.	1334	N.O.	N.O.	1565	1583
Hst-5 + Cu	1315	1334	1371	N.O.	1565	N.O.
Hst-5 + Zn + Cu	N.O.	1334	N.O.	N.O.	1565	1583
Hst-5 K13E/R22G	1315	1333	1371	N.O.	1561	N.O.
Hst-5 K13E/R22G + Zn	N.O.	1333	N.O.	N.O.	1561	1583
Hst-5 K13E/R22G + Cu	1315	1333	1371	N.O.	1561	N.O.
Hst-5 K13E/R22G + Zn + Cu	N.O.	1333	N.O.	N.O.	1561	1583
Hst-5 H18A/H19A	1315	1333	1371	N.O.	1561	N.O.
Hst-5 H18A/H19A + Zn	N.O.	1333	N.O.	N.O.	1561	1583
Hst-5 H18A/H19A + Cu	N.O.	1333	N.O.	N.O.	1561	N.O.
Hst-5 H18A/H19A + Zn + Cu	N.O.	1333	N.O.	N.O.	1561	1583
Peptide P	1317	1336	1370	N.O.	1564	N.O.
Peptide P + Zn	N.O.	1336	N.O.	1388	1564	1583
Peptide J	1317	1336	1370	N.O.	1564	N.O.
Peptide J + Zn	N.O.	1336	N.O.	1388	1564	1583
Peptide J/F	1321	N.O.	1369	N.O.	1567	N.O.
Peptide J/F + Zn	N.O.	1350	N.O.	1392	1555	N.O.
Cu/Zn SOD^42^	1320 (est., pD 9, 229 nm ex.)	1340 (est., pD 9, 229 nm ex.)	1360 (His-Zn, pD 9, 229 nm ex.)	1396 (His-Zn, pD 9, 229 nm ex.)	1564 (His-M, His61, pD 7.3, 229 ex.)	1580 (sh, est., pD 7.3, 229 ex.)

1352 cm^−1^ = N_τ_-Ligated His (Cu(II)-βAlaHis)^43^.

N.O., not observed.

To support these assignments, model compound data were acquired from histidine in D_2_O solution as a function of pD (Supporting Fig. S5). At pD 5 (Supporting Fig. S5A), histidine is cationic, with a protonated imidazolium cation side chain, a protonated amino terminus, and an anionic carboxylate at the carboxyl terminus. At pD 7.5 (Supporting Fig. S5B), histidine is zwitterionic with an unprotonated imidazole side chain. At pD 11 (Supporting Fig. S5C), histidine is anionic, with a deprotonated amino and imidazole group, and negative charge remaining on the carboxyl terminus. In D_2_O, the spectrum of the protonated imidazolium cation (pD 5) exhibits bands at 1408 and 1601 cm^−1^, while the spectrum of the deprotonated imidazole side chain exhibits new bands at 1317, 1370, 1479, and 1564 cm^−1^. These data are similar to results reported previously^42,43^. The normal mode assignments of the bands are: 1166 cm^−1^ in-plane ring modes mixing with N_1_-H^45^, 1323 cm^−1^ and 1351 cm^−1^ in-plane ring modes^45^, 1457 cm^−1^ ring N_1_-H^45^, 1484 cm^−1^ N-H in-plane bend of the protonated imidazole side chain^46^, 1568 cm^−1^ C=C stretching mode of the neutral imidazole sidechain in one tautomer^46^, 1577 cm^−1^ mixture of the in-plane ring mode with the N_1_-H^45^, 1585 cm^−1^ C=C stretch of the ring in one tautomer of histidine^46^, 1573–1588 cm^−1^ C_4_=C_5_ stretch when N_π_ coordinates a metal^47^, and 1594–1606 cm^−1^ C_4_=C_5_ stretch when N_τ_ coordinates a metal^47^.

Characteristic bands of tyrosine are also observed in the Hst-5 spectrum (Fig. 5IA,IIA) arising from Y10 and Y24. These bands are at 848 (not shown), 1176, 1210, and 1612 cm^−1^. To support the assignment to tyrosine, UVRR spectra were acquired from tyrosine and tyrosinate in H_2_O and D_2_O buffers (Supporting Fig. S6). At pH/pD 8.5 (Supporting Fig. S6A,B), the compound has a protonated phenolic ring, while at pH/pD 11, the aromatic ring is deprotonated (Supporting Fig. S6C,D). The contributions from the tyrosine and tyrosinate ring are resonantly enhanced at 244 nm. The highest energy ring stretching mode, Y8a, of the deprotonated phenolate is observed at 1602 cm^−1^ and is insensitive to the addition of D_2_O. Other characteristic bands are also D_2_O insensitive and are observed at 845, 1174, and 1207 cm^−1^ ^40,48^. At pH/pD 8.5, when the phenol ring is protonated, Y8a is observed at 1614 cm^−1^ in H_2_O and downshifts to 1610 cm^−1^ in D_2_O. In summary, model compound studies of tyrosine and histidine in solution support assignment of bands in the Hst-5 spectrum to vibrational contributions of the phenol/phenolate and imidazole side chains.

The UVRR spectra of the Hst-5 mutants, K13E/R22G (Fig. 5IB,IIIA) and H18A/H19A (Fig. 5IC,IVA), were obtained. The UV spectrum of each sample (Fig. 3) reveals that the extinction coefficient at 244 nm is similar in all and is approximately 1000 L mol^−1^ cm^−1^. These results are consistent with similar resonance enhancement factors in each sample. It has also been shown that the enhancement factors for imidazole bound to copper are similar to the unbound state^45^. A band at 1440 cm^−1^ is observed in each peptide spectrum. In D_2_O buffer, this band arises from amide II’ (C-N stretch) and is expected to be invariant to mutation^34^. Accordingly, the intensity of this band was used to normalize the spectra for comparison. After normalization, the spectrum of the K13E/R22G mutant is similar to that of wild type. In particular, the histidine and tyrosine bands have similar intensities and frequencies. On the other hand, the spectrum of the H18A/H19A mutant is distinguishable in the relative intensity of amide II’ to the 1313, 1371, and 1561 cm^−1^ bands, which arise from imidazole (Table 1). The H18A/H19A mutant also exhibits a 7 cm^−1^ shift of the Y8a tyrosine band, while a smaller and possibly not significant change is observed in the K13E/R22G mutant. The shift is assignable to a pK_a_ shift when the imidazole ring is changed to alanine. This change is accompanied by alterations in the 1179 and 1210 cm^−1^ bands, suggestive of hydrogen bonding and conformational changes at the tyrosines^40,48^.

### Effects of metal addition on the UVRR spectra

The effects of copper and zinc on the Hst-5 UVRR spectrum were evaluated. From previous studies of superoxide dismutase and a 27-mer zinc finger peptide, the expectation is that ligating histidine bands will shift in frequency (Fig. 1A and refs.^42,47,49,50^) when metal coordinates. Upon addition of Zn^2+^ (Fig. 5IIB) to the wildtype Hst-5 sample, the 1315 cm^−1^ band undergoes an upshift to 1334 cm^−1^, and the 1371 cm^−1^ band is decreased in intensity. In addition, the 1565 cm^−1^ band is reduced in intensity, and a zinc-induced shoulder is present at 1583 cm^−1^. Upon addition of Cu^2+^ (Fig. 5IIC), the spectral features are unaltered compared to the control (Fig. 5IIA), indicating that histidine does not provide coordination to copper in the wildtype. Upon addition of both Zn^2+^ and Cu^2+^ (Fig. 5IID), the spectral changes are similar to those observed with zinc alone. The results are consistent with coordination of zinc at a binding site that contains histidine. Interactions with copper are deduced to occur at a distinct, non-imidazole binding site. Shifts of the tyrosine Y8a band, from 1612 to 1605 cm^−1^ are also observed with zinc addition. These are attributed to electrostatic interactions. Note that while the UV spectrum of Hst-5 is not significantly altered by zinc addition (Fig. 3B), the spectrum is changed by the addition of copper (Fig. 3C,D). A similar set of zinc- and copper-induced changes was observed in the K13E/R22G mutant, indicating that the metal binding sites are relatively unchanged in this mutant. However, the H18A/H19A mutant shows a different pattern of spectral changes with metal addition. In the histidine mutant, addition of zinc shifts imidazole bands, as observed in wildtype, but this effect is no longer specific to zinc. Notably, in the mutant, copper and zinc have a similar effect on the spectrum. These results are attributed to loss of metal binding specificity in the H18A/H19A mutant.

### Modeling the conformationally dynamic site, metal addition to beta hairpin samples

The CD signal and thermal melting of the H18A/H19A variant are altered, when compared to wild type and the K13E/R22G mutant. We hypothesized that this result is due to a different distribution of conformers in the H18A/H19A mutant, which still provide a dynamic, low affinity zinc binding site. To test this hypothesis, we used UVRR spectroscopy to measure the effect of zinc addition on Peptide P, J, and J/F, which exhibit similar CD properties when compared to Hst-5.

**Figure 6:**
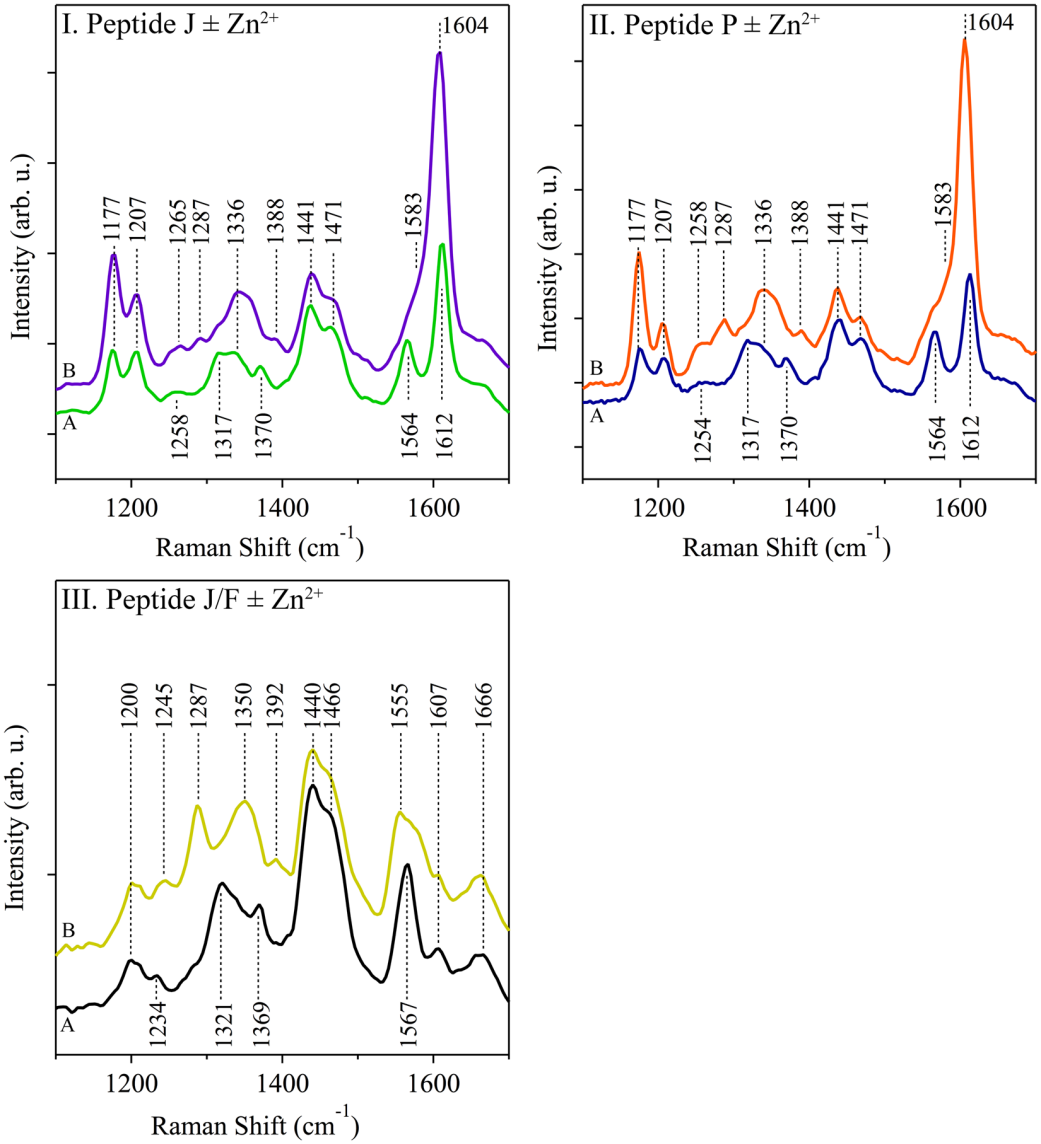
UVRR spectra of beta hairpin peptides. Samples: Peptide J (I) in the absence (A, green) or presence (B, purple) of equimolar ZnCl_2_; Peptide P (II) in the absence (A, blue) or presence (B, orange) of equimolar ZnCl_2_; Peptide J/F (III) in the absence (A, black) or presence (B, gold) of equimolar ZnCl_2_. The peptide concentration was 1 mM, and the samples contained equimolar ZnCl_2_ where noted. The buffer contained 5 mM HEPES, pD 7.5. Sample was recirculated using a peristaltic pump to prevent UV damage. Laser wavelength, 244 nm; laser power, 3.4 mW; scan time, 120 s; accumulations, 4. Data were averaged from at least two independent measurements (using at least two 1 mL samples). Tick marks denote 200 intensity units.

The UVRR spectrum of Peptide J/F was obtained (Fig. 6IIIA). This peptide contains no tyrosine side chains, but has a four histidine metal binding motif. Most of the spectral bands are readily assignable by comparison to the histidine model spectrum at pD 7.5 (Supporting Fig. S5). In the Peptide J/F UVRR spectrum, imidazole bands are observed at 1321, 1369, and 1567 cm^−1^ and are similar to bands observed in histidine solution at pD 7.5. Zinc was added at a one to one stoichiometry to Peptide J/F, and the UVRR spectrum was obtained (Fig. 6IIIB). At a one to one stoichiometry, a band at 1321 cm^−1^ decreases in intensity, and new bands appear at 1048 (not shown), 1200, 1287, 1350 cm^−1^. The band at 1567 broadens and shifts to 1555 and 1575 cm^−1^. UVRR spectra of Peptide P and J (Fig. 6I,II) exhibited similar imidazole bands and similar zinc induced shifts.

Peptide P and J contain a tyrosine side chain, and the UVRR spectra of these peptides exhibits bands assignable to Y5. Interestingly, the bands display zinc-induced shifts, similar to the results obtained with Hst-5. The tyrosine side chain is protonated at pD 7.5, and contributes to the spectrum at 848, 1177, 1207, and 1612 (Supporting Fig. S6). With zinc addition at a one to ratio (Fig. 6IB,IIB), a dramatic increase in intensity and small decrease in frequency for the ring stretching mode of tyrosine occurs. The tyrosinate band is now observed at 1604 cm^−1^ in the presence of zinc. The intensity increase is attributed to red shift observed of tyrosine UV absorption band, which increases resonance enhancement at 244 nm (Supporting Fig. S1). The frequency downshift is attributed to deprotonation and a change in the pK_a_ of tyrosine. The results are similar to those observed in Hst-5. Experiments were conducted to evaluate if the low affinity zinc binding site in the model peptides is specific to zinc. The site appears to be saturated at a 1:1 ratio in Peptide P. A stoichiometric ratio of 2:1 zinc to peptide instead of 1:1 did not lead to additional changes in the UVRR spectrum (Supporting Fig. S7). The UVRR spectra reflected no significant change when manganese or calcium are added to a Peptide J sample in H_2_O buffer (Supporting Fig. S8). Also note that modification of the sequence to contain the four histidine or three histidine-aspartate binding site is necessary to observe these spectral changes. The addition of zinc to Peptide A, which contains the cross strand Y5-H14 pair, has no significant effect on the spectrum in H_2_O buffer (Supporting Fig. S9).

## Discussion

The histatins play an important role in human health. The oral cavity presents a site for opportunistic infections. Antimicrobial immune peptides, like Hst-5, differ in concentrations in some immunocompromised individuals. It has been shown that oral fungal infection by *Candida albicans* is increased in an HIV-positive population that also exhibited a lower salivary Hst-5 concentration^51^. Additionally, it has been observed by proteomic analysis that patients with primary and secondary Sjögren’s syndrome have lower levels of identifiable salivary histatins^52^. The Hst-5 K13E/R22G and Hst5 H18A/H19A mutants significantly decrease candidacidal activity compared to wild type^9,10^. Binding of nickel, zinc, and copper is proposed to influence the activity of Hst-5. While zinc and copper are present in blood at a concentration of approximately 1 ppm^53^ in saliva, zinc is present at approximately 0.1–0.2 ppm^53,54^, and copper has been measured to be approximately 2–100 ppb^54^. Incubation with metal ions increases the antifungal properties of the peptide^28^. Ni^2+^ is also believed to be coordinated by Hst-5, though the proposed binding sites for Ni^2+^ compete with the proposed binding sites for both Zn^2+^ and Cu^2+^. Hst-5 could possibly be relevant in allergic reactions to nickel^29^.

Here, we report CD and UVRR studies of Hst-5 and two sequence variants. We find that the addition of zinc to wild type Hst-5 stabilizes the peptide against thermal denaturation. Further, frequency shifts are observed in characteristic Raman bands when zinc is added to wild type Hst-5. These bands are assigned to the imidazole side chain by comparison to spectra of the amino acid in solution and to a family of conformationally dynamic beta hairpins. The H18/H19 mutant exhibits altered behavior with CD and thermal melting, when compared to wild type. This change is consistent with an alteration in the distribution of stable conformers in the mutant. In this H18/H19 and the K13E/R22G mutant, UVRR spectroscopy reveals an electrostatic perturbation of tyrosine, which is evident as a downshift of the highest energy ring stretching mode. This effect is also reproduced in tyrosine-containing, zinc binding beta hairpins. We find that the H18A/H19A mutant loses metal specificity and appears to bind copper in an imidazole-containing binding site.

Three models have been proposed for the bioactivity of histatins^7–15^. In one, histatins interrupt fungal mitochondrial electron transfer. In a second, histatins generate reactive oxygen species, which is toxic and antimicrobial. In a third, histatins act as membrane permeant peptides and disrupt the electrochemical gradient. Based on our results, the change in antifungal activity in H18A/H19A is most likely due to change in stability of the peptide and a loss of metal specificity.

Previous studies have been conducted on Hst-5 (reviewed in ref.^7^) and the two mutants Hst5 K13E/R22G and Hst5 H18A/H19A. The structure of wild type Hst-5 has been studied by NMR in trifluorethanol/water and DMSO/water mixtures^17,18^. In these hydrophobic environments, the peptide adopts a helical conformation, though the peptide is considered to be disordered in purely aqueous solvent. Isothermal titration calorimetry has identified at least one binding site for Zn^2+^ and one for Cu^2+^. The measured binding constants for the wild type peptide for Zn^2+^ and Cu^2+^ are 10^−5^ and 10^−7^ M, respectively^55^. NMR experiments on Hst-5 in aqueous solvent in the presence of three equivalents of Zn^2+^ revealed a broadening of three histidine peaks along with one glutamate peak. In the presence of three equivalents of Cu^2+^, the reduction in intensity of an aspartate resonance was apparent^26^. Mass spectrometry characterization of Hst-5 has revealed information regarding the stoichiometry of metal-binding^37^. At pH 7.5, a 2:1 ratio results in 73% relative abundance of a dicopper-Hst5 complex and 27% monocopper-Hst-5^37^. For the case of 2:1 Zn^2+^:peptide samples, only 22% of the sample was found to bind Zn^2+^, and the stoichiometry of this complex was 1:1^37^. In sample preparations with higher Zn^2+^:peptide ratios, a greater percentage of the sample was found to bind at least one equivalent of Zn^2+^ ^37^. This previous work also investigated the CD spectra of Hst-5 in the presence of 5 equivalents of Zn^2+^ and Cu^2+^ at 25 °C, pH 7.2. In the presence of either Cu^2+^ or Zn^2+^, the CD spectrums was altered, although the decrease in magnitude of the CD signal, noted here with zinc addition, was not observed^37^.

The sequences of the beta hairpin peptides used here were inspired by the zinc binding sites in carbonic anhydrase^56^ and superoxide dismutase^57^. Zinc binding proteins are ubiquitous in biology and can be divided into two main classes^58^. In structural zinc proteins, such as the zinc finger, the divalent ion is coordinated by cysteine and zinc^59^. In catalytic zinc proteins, such as carbonic anhydrase^56^ and superoxide dismutase^57^, zinc coordination involves histidine, aspartate, and active site water molecule. Peptide mimics of zinc binding peptides have been described previously, based on zinc finger cysteine/histidine motifs^60,61^. For example, CP-1 has a hydrophobic core and has a dissociation constant of 10^−7^ for Co^2+^ and 10^−12^ for Zn^2+^ ^60^. Peptide mimics of carbonic anhydrase, based on ZnN_3_O coordination, have also been constructed^62,63^. Recently, it has been shown that heptapeptides can form zinc-containing amyloid fibrils and catalyze the hydrolysis of esters, in a manner similar to carbonic anhydrase^64,65^. The K_M_ was 1.8 mM^64^. A similar strategy has been used to introduce a specific zinc binding site into a 16-mer beta hairpin^66^ and an octapeptide^67^. Zinc coordination was verified by metal-induced changes in the CD spectrum and by NMR spectroscopy^66,67^.

Previous studies of Cu/Zn containing superoxide dismutase (SOD) and its relevant model compounds have shown that D_2_O exchange intensifies UVRR bands of histidine and makes them more easily detectable at 229 nm^42,49^ and 240 nm^50^. In SOD, the Cu ion is coordinated by four histidine residues, and the Zn ion is coordinated by three histidine residues and one aspartic acid residue. Among the coordinated histidines, His61 has a unique imidazolate structure (His-) and bridges between copper and zinc^68,69^. The UVRR spectrum of SOD reveals characteristic metal-induced changes in bands assignable to Cu-His, Zn-His, and the bridging His61^68^.

In the case of SOD wild type, the bridging imidazolate His61 has been assigned to the UVRR bands observed at 986 cm^−1^, 1050 cm^−1^, 1282 cm^−1^, 1292 cm^−1^, and 1564 cm^−1^ ^42^. In the apoenzyme, these frequencies are not observed^42^. Additional histidine-zinc vibrational bands at 1360 cm^−1^ and 1396 cm^−1^ are perturbed in the apoenzyme as well^42^. Histidine-metal bands at approximately 1340 cm^−1^ are downshifted to approximately 1320 cm^−1^ in the apocomplex^42^. The normal mode assignments have been described previously^70^. Imidazolium-d_0_ exhibits a ring expansion and N-H wag at 1322 cm^−1^ and a C^(+)^N + C-N stretch at 1590 cm^−1^ ^70^. Imidazole-d_0_ modes include C_2_N_1_ + C_5_N_1_ stretch + N-H wag at 1578 cm^−1^ ^70^. A C-H wag is calculated to occur at 1394 cm^−1^ for imidazole-d_0_ ^70^. Perturbation of the frequency or intensity of these histidine ring modes is consistent with metal-binding and is observed when wild type and apo SOD are compared. In SOD, there is a single tyrosine Y108, which has an elevated pK_a_ when metal is bound. A shift of the Y8a band was reported with metal binding^42^.

While intrinsically disordered proteins are ubiquitous (reviewed in refs.^1,2,71^), new paradigms are needed to link structure and function in these proteins. To model and interpret the histatin results on disordered Hst-5, we employed the family of designed beta hairpin peptides, which were engineered to provide a binding site for zinc. In beta hairpins, the formation of the antiparallel beta structure is spontaneous^72^. Beta hairpins are dynamic in solution and sample an ensemble of structural states^35,73,74^. When the samples are treated with zinc, a change in the UVRR spectrum is observed, which is similar to the change observed in the histatin peptides. In addition, a change in vibrational spectrum of an adjacent tyrosine is observed, which is consistent with a decrease in its pK_a_ and the observed red shift of its electronic spectrum. The UVRR bands are similar to the Hst-5 bands observed here.

In summary, the results presented here provide new information concerning metal binding sites in intrinsically disordered peptides. A combination of spectroscopic studies of the biological sample and peptide models provides a robust framework for interpretation. The results support the conclusion that Hst-5 is a conformationally dynamic peptide, with bioactive forms that are preferentially stabilized by zinc interactions. The stability of the peptide is responsive to the addition of divalent metal ions, and zinc binds to histidine residues in the peptide. The copper and zinc sites are distinct, and a loss of specificity and conformational destabilization are associated with a decrease in antimicrobial activity. This work provides a basis for design of conformationally disordered, chemically activated peptides, which could be used to treat disease and enhance immunity.

## Methods

The 18-mer peptides, Peptides P, J, and J/F were synthesized by solid-state synthesis and were obtained from Genscript, USA, Inc (Piscataway, NJ) or New England Peptide (Gardner, MA). The sequences are: Peptide A, IMDRYRVRNGDRIHIRLR; Peptide P, IHDHYRVRNGDRIHHRHR; Peptide J, IDDHYRVRNGDRIHHRHR; Peptide J/F, IDDHFRVRNGDRIHHRHR. Histatin-5 (Hst-5) and the two double mutants, K13E/R22G and H18A/H19A, were synthesized by solid-state synthesis and were obtained from Genscript, USA, Inc (Piscataway, NJ). The sequences are Hst-5, DSHAKRHHGYKRKFHEKHHSHRGY; Hst-5 K13E/R22G, DSHAKRHHGYKREFHEKHHSHGGY; Hst-5 H18A/H19A, DSHAKRHHGYKRKFHEKAASHRGY. Mass spectrometry was used to verify the molecular weights of the peptides. The molecular weights matched the theoretical prediction from the sequence. See Supporting Information for more details and a description of the methods used for UV-Vis, CD, and UVRR spectroscopy.

## Supplementary information

Supplementary information

## Data Availability

The data are available for review upon request.
