# Supplementary material for: Specific metallo-protein interactions and antimicrobial activity in Histatin-5, an intrinsically disordered salivary peptide
Source: Sci Rep. 2019 Nov 21;9:17303. doi: 10.1038/s41598-019-52676-7 (PMC6872563; doi:10.1038/s41598-019-52676-7)
Supplement: Supplementary file 1 — Supplementary information [file 41598_2019_52676_MOESM1_ESM.pdf]

Supporting Information for:

Specific metallo-protein interactions and antimicrobial activity in Histatin-5, an intrinsically disordered salivary peptide

Tyler G. McCaslin,[1,2] Cynthia V. Pagba,[1,2] Jiby Yohannan,[1,2] and Bridgette A. Barry[1,2]*

[1]School of Chemistry and Biochemistry, 901 Atlantic Drive NW and

[2]The Parker H. Petit Institute of Bioengineering and Bioscience

Georgia Institute of Technology, Atlanta, GA 30332, U. S. A.

*Email: bridgette.barry@chemistry.gatech.edu, Phone: 404-385-6085

Experimental Methods

Materials. Peptide samples were suspended either in a H_2O or D_2O buffer containing 5 mM 2-[4-(2-hydroxyethyl)piperazin-1-yl]ethanesulfonic acid (HEPES), pH/pD 7.5, to give 1 mM solutions. Acetic acid (VWR, Radnor, PA)/sodium acetate (Thermo Fisher Scientific, Inc., Waltham, MA) and sodium borate (Mallinckrodt, Paris, KY)/NaOD were used to make buffers at pD 5 and pD 11, respectively. The pH/pD was adjusted using NaOH or NaOD. HEPES, histidine, and tyrosine were purchased from Sigma-Aldrich (St. Louis, MO), and D_2O and NaOD were purchased from Cambridge Isotopes (Tewksbury, MA, 99.9% D). The pD is reported as the uncorrected meter reading, because the small solvent isotope shift effects on weak acid pK_a values are compensated by the D_2O-induced change in response of the glass pH electrode[1]. In some experiments, $ZnCl_2$ (Alfa Aesar, Tewksbury, MA), $CuCl_2$ (Alfa Aesar, Tewksbury, MA), $MnCl_2$ (ICN Biomedicals, Aurora, OH), $CaCl_2$ (EMD Millipore, Billerica, MA) were used in buffered solutions.

UV-Vis Spectroscopy. UV-Vis absorption spectra were recorded on a Shimadzu UV 1700 spectrometer. The slit width was 1 nm, the resolution was 1 nm, and the scan speed was 6.5 nm/s. The peptide concentration was 500 μM. The zinc concentration ranged from 500 μM (1:1 Peptide to Zn ratio) to 4 mM (1:8 Peptide to Zn ratio). The buffer contained 5 mM HEPES, pH 7.5 or pD 7.5. The spectra were averaged from two independent measurements.

Circular Dichroism (CD). A Jasco J-810 CD spectropolarimeter equipped with a Peltier-type cell was employed. Spectra were collected from 250 nm to 193 nm in 1 mm quartz cells. Eight accumulations per scan were averaged in three to nine independent measurements for each of the conditions. Parameters used were: sensitivity, 100 mdeg; data pitch, 2 nm; scan speed, 50 nm/min; response time, 1 s; bandwidth, 1 nm.

UVRR Spectroscopy. The spectra were obtained at room temperature using a 244 nm (3.4 to 3.7 mW) probe beam.[2-3] The probe beam was generated from an intracavity frequency-doubled Argon ion laser (Cambridge LEXEL 95, Fremont, CA) and was coupled to a Raman microscope system (Renishaw inVia, Hoffman Estates, IL) equipped with UV-coated, deep depletion charge-coupled device. The spectral resolution was 6 cm^{-1} and the separation between the data points was 3.8 cm^{-1}. The peak positions are reported to a precision of ± 2 cm^{-1}. Peptide samples (1 mL) were recirculated using a peristaltic pump through a nozzle (~120 μm inner diameter) to prevent sample damage due to UV irradiation. The flow rate was 4.5 mL/min, and the pump was plumbed with silicone tubing. The spectra were averaged from at least two independent measurements.

References

1 Jenson, D. L. & Barry, B. A. Proton-coupled electron transfer in photosystem II: proton inventory of a redox active tyrosine. *J. Am. Chem. Soc.* **131**, 10567-10573 (2009).

2 Pagba, C. V. & Barry, B. A. Redox-induced conformational switching in photosystem-II-inspired biomimetic peptides: a UV resonance Raman study. *J. Phys. Chem. B* **116**, 10590-10599 (2012).

3 Chen, J. & Barry, B. Ultraviolet resonance Raman microprobe spectroscopy of photosystem II. *Photochem. Photobiol.* **84**, 815-818 (2008).

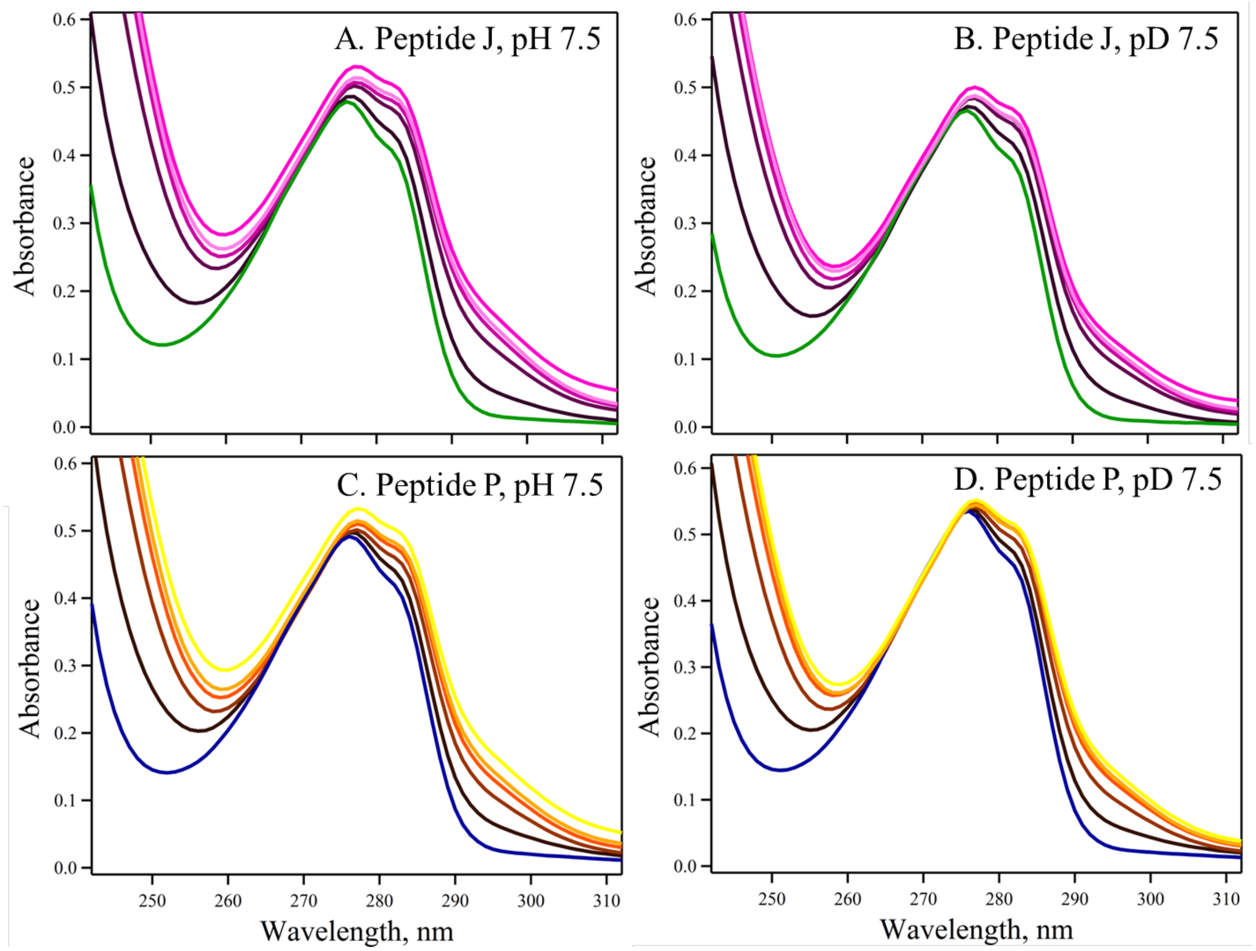

Figure S1. UV absorption spectra of Peptide J (A and B) and Peptide P (C and D) with increasing concentrations of Zn^{2+} (500 µM, dark purple and dark brown; 1mM, purple and brown; 2 mM, light purple and orange, 3 mM, pink and light orange; 4 mM, light pink and yellow) at pH 7.5 (A and C) and pD 7.5 (B and D). The analyte concentration was 500 µM. The buffer contained 5 mM HEPES. The spectra were averaged from two independent measurements.

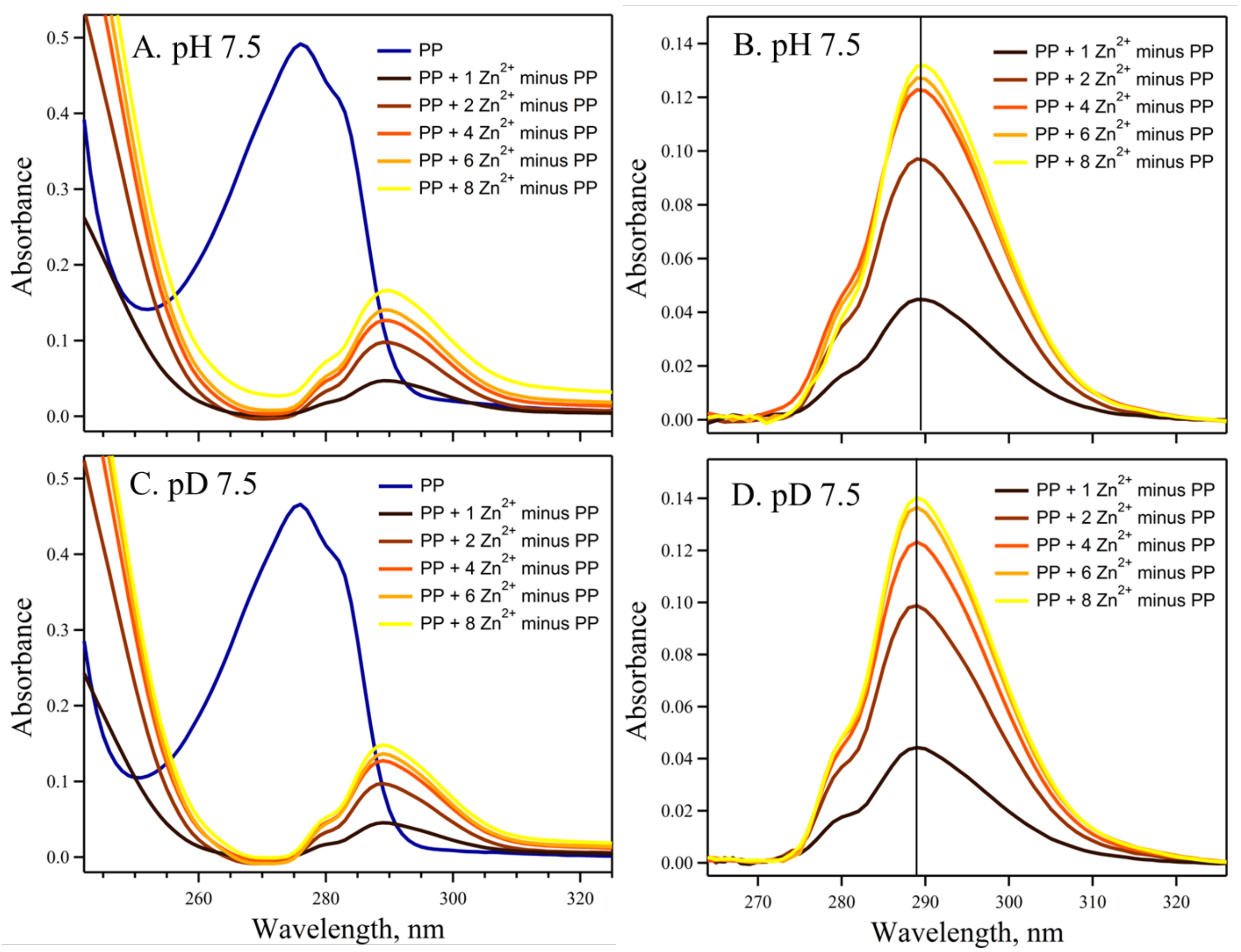

Figure S2. UV absorption spectra of Peptide P (A and C) (green) and difference absorption spectra obtained by subtracting the Peptide P spectrum from that of Peptide P and Zn^{2+} solution with variation in the Peptide:Zn^{2+} stoichiometry ratios, 1:1 (dark brown), 1:2 (brown), 1:4 (orange) and 1:6 (light orange), 1:8 (yellow). The difference spectra (baseline corrected) are expanded in panels B and D. The measurements were performed at pH 7.5 (A and B) and pD 7.5 (C and D). The peptide concentration was 500 μM. The buffer contained 5 mM HEPES. The spectra were averaged from two independent measurements.

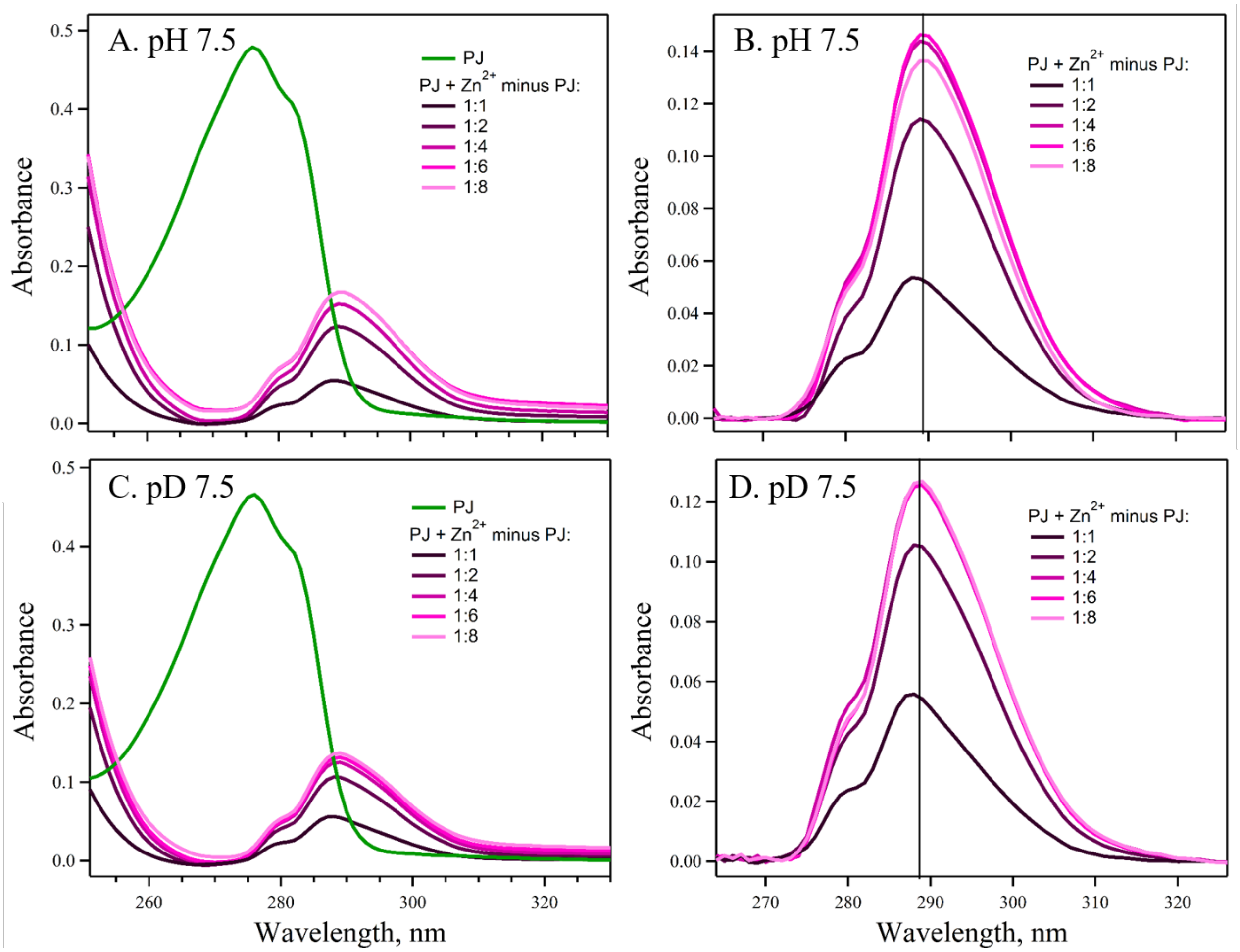

Figure S3. UV absorption and difference spectra of Peptide J (green) at pH 7.5 (A) and pD 7.5 (C). Difference spectra, showing the effects of zinc addition, were obtained by subtracting the Peptide J spectrum from that of a Peptide J and Zn^{2+} mixture (A, pH 7.5 and C, pD 7.5). The ratios were 1: 1 (dark purple), 1:2 (purple), 1:4 (light purple), and 1:6 (pink), 1:8 (light pink). The difference spectra are expanded in panels B and D. The analyte concentration was 500 µM, and the buffer contained 5 mM HEPES, pL 7.5. The spectra were averaged from two independent measurements.

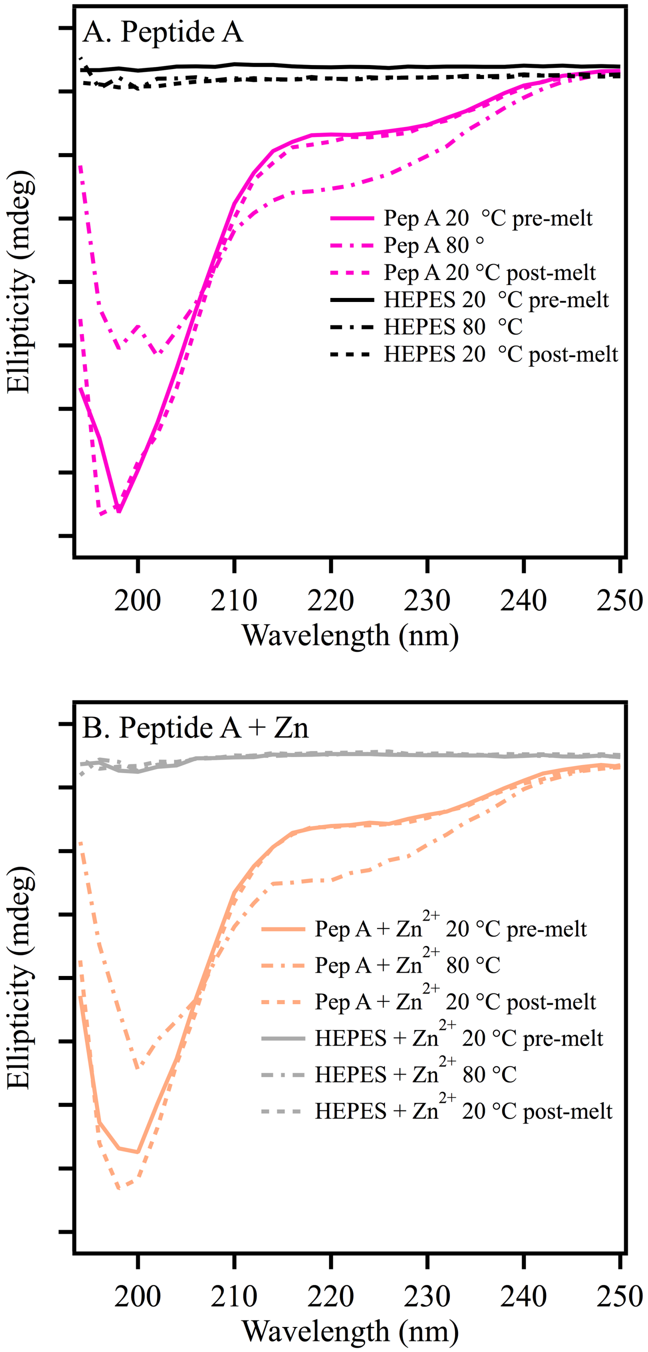

Figure S4. CD spectra of Peptide A (A, pink) and buffer (A, black) and Peptide A (B, peach) and Zn^{2+} and buffer (B, grey). Spectra were acquired at 20 °C (solid trace), then the samples were heated in the Peltier cell at 80 °C (dot-dashed trace), and subsequently cooled back to 20 °C (dashed trace). Tick marks denote 4 mdeg. Spectra were averaged from three replicates. The peptide concentration was 100 µM, and equimolar $ZnCl_2$ was added where noted. The buffer contained 5 mM HEPES pH 7.5. Negative controls acquired from HEPES buffer or HEPES buffer plus zinc (no peptide) are shown in A and B in black and grey.

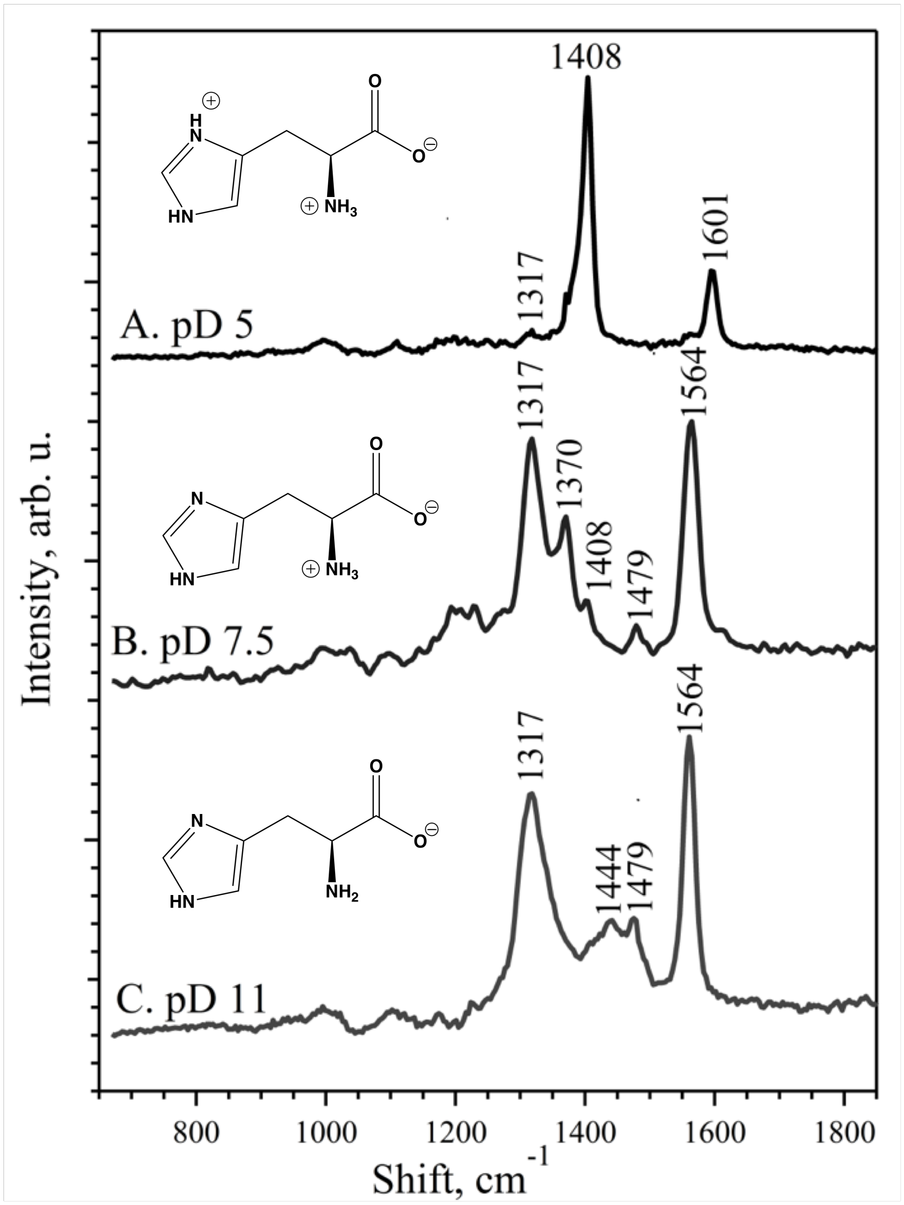

Figure S5. UV Raman spectra of 5 mM Histidine at (A.) pD 5, (B.) pD 7.5 and (C.) pD 11 The buffer contained 5 mM of acetate (pD 5), HEPES (pD 7.5) and borate (pD 11). The spectra are normalized with respect to most intense peak for each sample. Each tick mark is 0.1 unit.

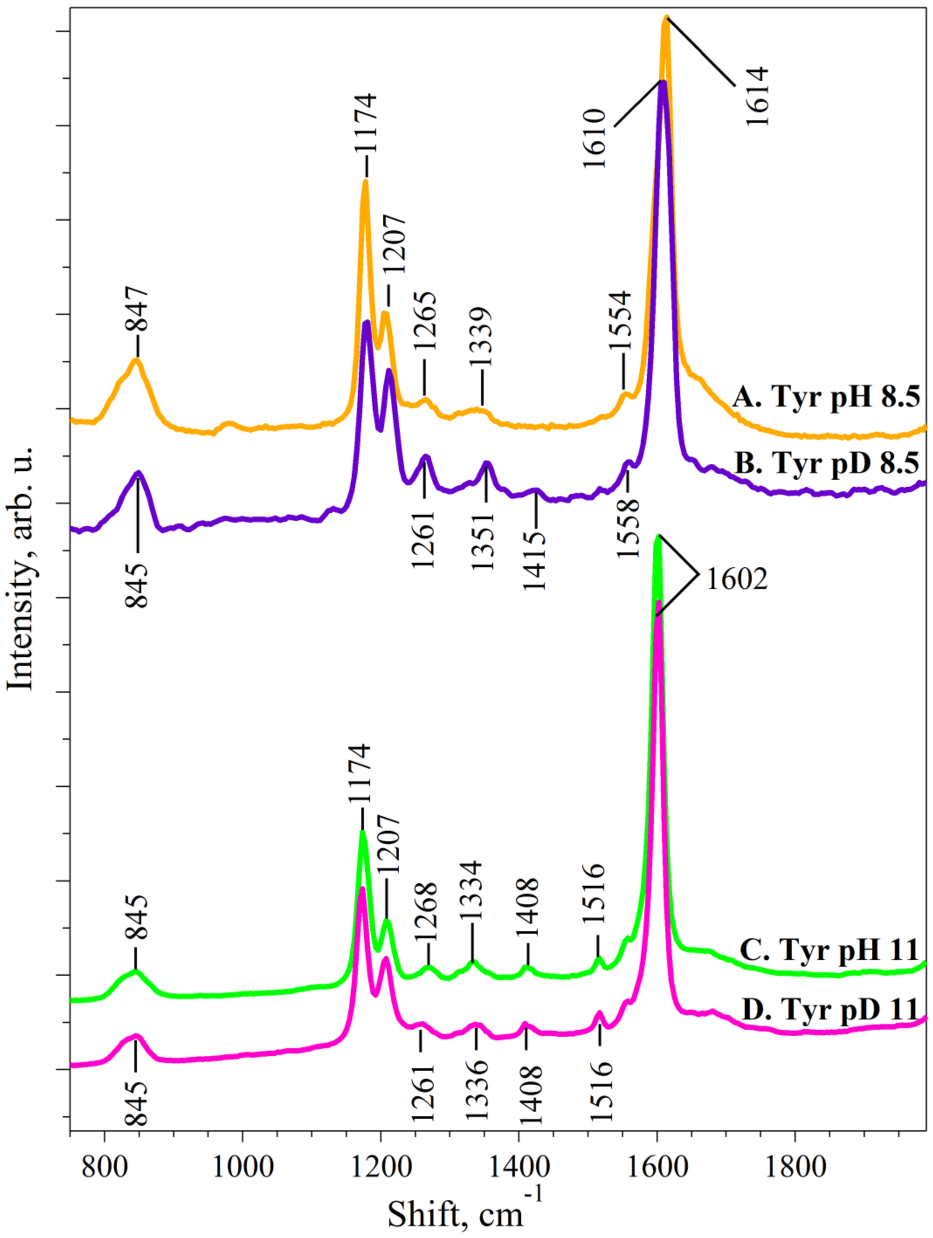

Figure S6. UV Raman spectra of 1 mM tyrosine at (A.) pH 8.5, (B.) pD 8.5, (C.) pH 11 and (D.) pD 11. The spectra are normalized with respect to the most intense peak. Each tick mark is 0.05 unit. The buffer contained 5 mM of TAPS (pL 8.5) or 5 mM borate (pL 11).

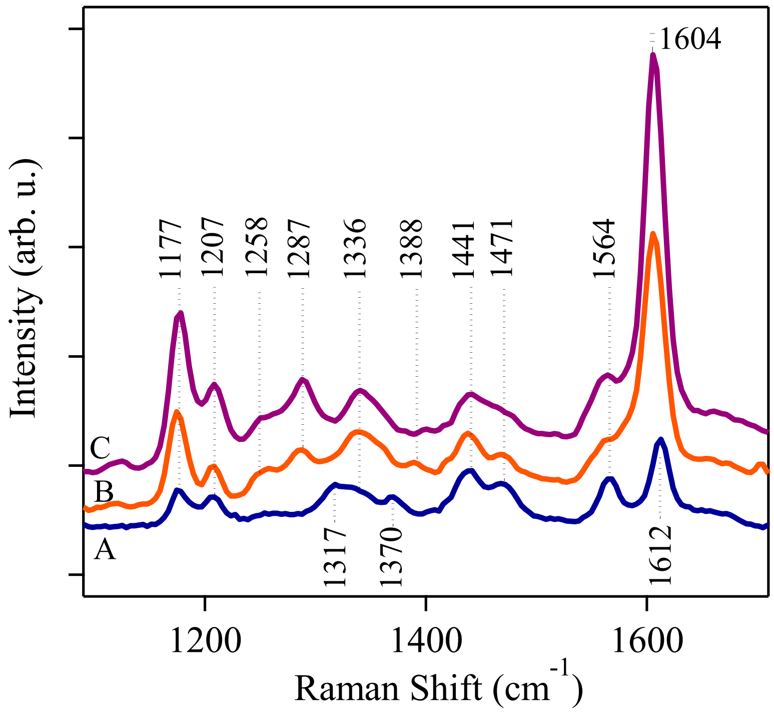

Figure S7. UVRR spectra of Peptide P after the addition of two different concentrations of $ZnCl_2$. Samples: 1 mM Peptide P alone (A, blue), Peptide P with 1 mM $ZnCl_2$ (B, orange), and Peptide P with 2 mM $ZnCl_2$ (C, purple) . The buffer contained 5 mM HEPES pD 7.5. The sample was recirculated using a peristaltic pump to prevent UV damage. Laser wavelength, 244 nm; laser power, 3.4 mW; scan time, 120 s; accumulations, 4. Data were averaged from at least two independent measurements. The tick marks (y-axis) denote 500 units.

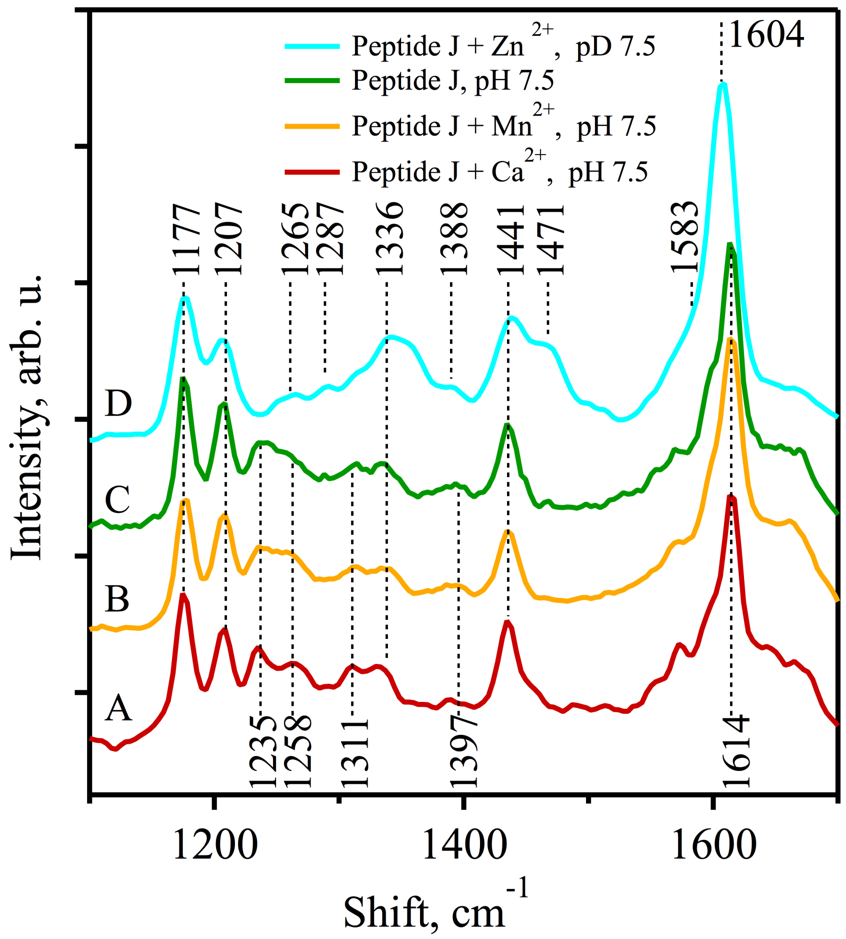

Figure S8. UVRR spectra of Peptide J and Ca^{2+} (A, red), Peptide J and Mn^{2+} (B, light orange) and Peptide J (C, green) at pH 7.5. In (D), Peptide J and Zn^{2+} pD 7.5 is reproduced from Figure 6 in the main text. In A-C, the peptide concentration was 1 mM, when present, the calcium and manganese concentrations were 5 mM, and the buffer contained 5 mM HEPES in H_2O (pH 7.5). In (D), the peptide concentration was 1 mM, and the sample contained equimolar Zn^{2+}. The data were averaged from at least two independent measurements. Tick marks denote 200 intensity units.

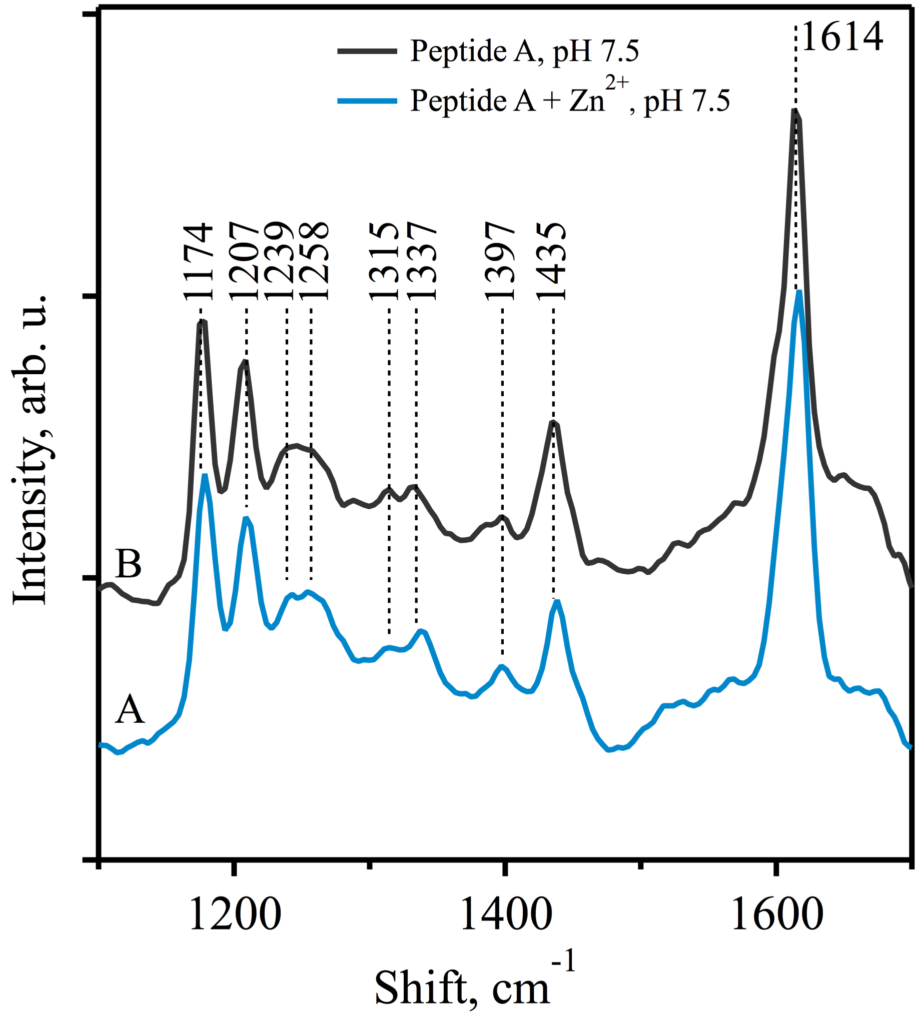

Figure S9. UVRR spectra of Peptide A in the presence (A, blue) or absence (B, black) of Zn^{2+} at pH 7.5. The peptide concentration was 1 mM, and the zinc concentration was 5 mM. The buffer contained 5 mM HEPES in H_2O (pH 7.5). The data were averaged from at least two independent measurements. Tick marks denote 200 intensity units.
